# Regarding the case image article entitled “A case of angle‐closure glaucoma caused by spontaneous lens dislocation” by Kondo et al.

**DOI:** 10.1002/ccr3.8755

**Published:** 2024-04-09

**Authors:** Mehrdad Motamed Shariati

**Affiliations:** ^1^ Eye Research Center Mashhad University of Medical Sciences Mashhad Iran

I am writing to commend the authors on their valuable contribution to the field of ophthalmology research through their case image article entitled “A case of angle‐closure glaucoma caused by spontaneous lens dislocation”,^1^ which was published in the Clinical Case Reports journal on December 5, 2022. The study addresses a rare and unusual condition called spontaneous crystalline lens dislocation (CLD). The authors found the CLD in the orbital axial CT‐scan image. However, I wish to bring to your attention a potential interpretation error in the study that could impact the accuracy of the reported results.

Ectopia lentis is defined as the malposition of the crystalline lens, which could be partially displaced or completely dislocated outside the patellar fossa. Regarding the etiology, CLD is divided into two major categories: congenital and acquired.^2^ The pathogenesis is the dysfunction of the zonular fibers. Spontaneous CLD is an extremely rare condition. Pseudoexfoliation (PEX) is the most common cause of zonular weakness in adults. However, hypermature cataract, longstanding retinal detachment, and high myopia are among the reported risk factors. Depending on whether the CLD occurs anteriorly or posteriorly, different clinical symptoms and treatment approaches will be required.^3^, ^4^ Anterior CLD usually leads to angle colure glaucoma, while posteriorly displaced crystalline lens causes ocular inflammation and visual loss (Figure 1).

**FIGURE 1:**
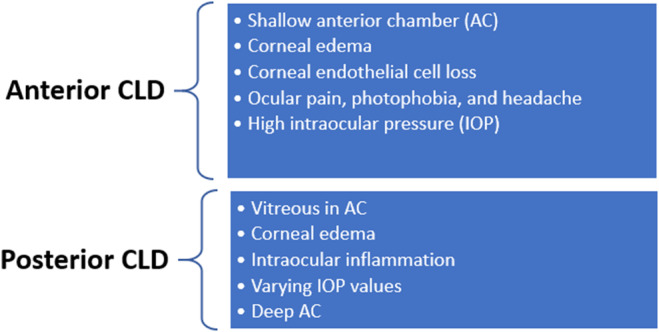
Clinical signs and symptoms in CLD. CLD, crystalline lens dislocation.

As the authors showed in Figure 1 of the original article, the patient's crystalline lens is dislocated posteriorly into the vitreous cavity. This condition usually leads to intraocular inflammation and visual loss. Angle‐closure is improbable in posterior CLD. Besides, the big size and globular shape of the crystalline lens in the mentioned image suggest the existence of the cataract. Usually, non‐traumatic cataracts develop slowly over time, and the patient probably had vision loss before the occurrence of the CLD. However, we know nothing about the ocular risk factors of spontaneous CLD including PEX in this patient.

## AUTHOR CONTRIBUTIONS

**Mehrdad Motamed Shariati:** Conceptualization; investigation; writing – original draft; writing – review and editing.

## FUNDING INFORMATION

The authors received no funding.

## CONFLICT OF INTEREST

The authors declare that they have no competing interests.

## CONSENT

Not applicable.

## Data Availability

Data sharing not applicable to this article as no datasets were generated or analysed during the current study.
